# Retrospective cohort study of the microbiology and clinical outcomes of spontaneous bacterial peritonitis (SBP) within a US academic health system

**DOI:** 10.1017/ash.2025.10203

**Published:** 2025-10-30

**Authors:** P. Christopher Parish, Alex D. Taylor, Tyler J. Stone, Vera P. Luther, Christopher A. Ohl, Michael E. DeWitt, James R. Beardsley

**Affiliations:** 1 Department of Pharmacy, Atrium Health Wake Forest Baptist, Winston-Salem, NC, USA; 2 Department of Internal Medicine, Section on Infection Diseases, Wake Forest University School of Medicine, Winston-Salem, NC, USA; 3 Department of Biology, Wake Forest University, Winston-Salem, NC, USA

## Abstract

**Objective:**

To determine the local applicability of 2021 American Academy for the Study of Liver Diseases spontaneous bacterial peritonitis (SBP) treatment guidelines by evaluating the microbiology and clinical outcomes of SBP cases in an academic health system.

**Design:**

Retrospective cohort study.

**Setting:**

Five-hospital academic health system.

**Patients:**

Hospitalized adult patients with SBP.

**Methods:**

This study involved 2 components. First, patients meeting inclusion criteria with peritoneal fluid cultures positive for a pathogen were included in the culture-positive group. Antibiotic susceptibilities were analyzed for these patients. Second, remaining culture-negative patients were randomly selected and sequentially evaluated until the culture-negative and culture-positive groups were approximately the same size. Clinical data for all patients were evaluated based on empiric antibiotics received.

**Results:**

Forty-nine patients with culture-positive SBP and 48 patients with culture-negative SBP were included. Eight (16%) positive cultures contained TGC-nonsusceptible organisms. Patients receiving empiric third-generation cephalosporin (TGC) monotherapy had similar clinical outcomes as patients receiving empiric broad-spectrum therapy, including similar 30-day mortality (36% vs 38%; *P* = 1.00), 90-day mortality (55% vs 55%; *P* = 1.00), and median duration of hospitalization (6.5 d vs 8 d; *P* = .25). ICU admission, recent hospitalization, and nosocomial infection were not associated with TGC-nonsusceptible pathogen isolation in a univariate logistic regression analysis.

**Conclusions:**

Within our health system, 16% of isolates in culture-positive SBP patients were nonsusceptible to TGCs. No statistically significant difference was detected in clinical outcomes in patients receiving TGC or broader-spectrum antimicrobial therapy.

## Introduction

Spontaneous bacterial peritonitis (SBP) is a common infection in patients with cirrhosis and is associated with 30-day mortality rates around 25%.^1,2^ The American Association for the Study of Liver Diseases (AASLD) published consensus guidelines in 2012 for the management of SBP.^3^ These guidelines recommended third-generation cephalosporins (TGCs), including ceftriaxone or cefotaxime, for the empiric treatment of SBP, citing an infection resolution rate of >90%.^3^ Recently, the appropriateness of empiric TGC therapy for SBP has been questioned, with updated consensus guidelines published by the AASLD citing increasing reports of SBP caused by multidrug-resistant organisms (MDROs).^4^ Factors associated with an elevated risk for SBP caused by a MDRO include patients who are admitted to intensive care units (ICUs), have nosocomial infections, or were recently hospitalized.^4–6^ Guideline recommendations now suggest empiric therapy with anti-pseudomonal beta-lactams, including meropenem, in addition to methicillin-resistant *Staphylococcus aureus* (MRSA) coverage in select patients with MDRO risk factors.

The data cited in support of these recommendations were primarily derived from centers with high rates of MDROs.^4^ A small, prospective study conducted in Italy compared broad versus narrow spectrum antimicrobial therapy for SBP and showed superior clinical outcomes in patients empirically treated with daptomycin and meropenem in comparison to TGC monotherapy.^5^ However, the SBP microbiology in the study was composed almost entirely of TGC-resistant organisms (81%).^5^ A multicenter, retrospective evaluation of SBP outcomes in South Korea showed that patients with severe clinical presentations had superior clinical outcomes if treated empirically with a carbapenem.^6^ However, this analysis did not include a microbiological evaluation of peritoneal cultures to assess causative organisms.^6^

Considering these limitations in the published literature, the objective of this study was to determine if revised guidelines which recommended broader-spectrum therapy would benefit the patients in our health system. The study objective was addressed through two complementary approaches. First, the prevalence of MDRO organisms from culture-positive SBP cases was determined. Second, a retrospective cohort study was performed to evaluate clinical outcomes of SBP patients receiving empiric TGC therapy versus broad-spectrum empiric therapy.

## Methods

This was an institutional review board-approved, retrospective, observational study evaluating SBP patients. The study evaluated patients admitted between January 2010 and December 2022 to a five-hospital academic health system with over 1 500 beds located in Central North Carolina. A query of electronic health records identified patients ≥ 18 years old with an ICD-9 or ICD-10 code for SBP and liver dysfunction who were admitted to this health system during the study period. The peritoneal fluid culture results from these patients were evaluated to categorize patients with culture-positive and culture-negative SBP. Patients were included in the culture-positive group if they had a peritoneal fluid culture positive for a pathogen and a concomitant peritoneal fluid polymorphonuclear (PMN) cell count > 250 cells/mm.^3^ Isolates were not considered to be pathogenic if they were a coagulase-negative *Staphylococcus* species or if the isolate was determined to be a contaminant as documented by the patient’s treatment team in the medical record. The remaining culture-negative patients were randomly selected and sequentially evaluated until the culture-negative and culture-positive groups were approximately the same size. Culture-negative patients were defined as having a PMN cell count > 250 cells/mm^3^ in the absence of a peritoneal culture positive for a pathogen. Patients were excluded from both the culture-positive and culture-negative groups if they had concomitant secondary peritonitis based on the documentation of the treating clinician. Patients were also excluded if they did not receive empiric antimicrobial therapy with a TGC (ceftriaxone or cefotaxime) or broader-spectrum therapy, defined as an antimicrobial regimen with additional gram-positive coverage (ie, vancomycin, linezolid, or daptomycin) or gram-negative coverage (ie, cefepime, ceftazidime, piperacillin/tazobactam, carbapenems) compared to TGCs.

The baseline characteristics that were collected included age, sex, components of the MELD-Na score,^7^ etiology of cirrhosis, and SBP prophylaxis at admission, and the following previously reported risk factors for needing broad-spectrum therapy: admission to an ICU, previous hospitalization within 90 days, and nosocomial SBP, defined as collection of a peritoneal fluid sample that was diagnostic for SBP (PMN count > 250 cells/mm^3^) at least 48 hours after hospital admission. The first aim of the study was to establish the prevalence of MDRO organisms at the study site, which was calculated as the percentage of patients in the culture-positive SBP cohort with a ceftriaxone-nonsusceptible pathogen isolated from peritoneal fluid culture. The definition of nonsusceptibility included isolates with a ceftriaxone minimum inhibitory concentration (MIC) > 1 μg/mL or those not tested because of intrinsic resistance (eg, *Enterococcus* spp.). The primary end point of the cohort study portion of our investigation was 30-day mortality comparing patients who received empiric TGC versus broader-spectrum therapy. Secondary endpoints included 90-day mortality, length of stay, and broadening of antimicrobial therapy by clinical teams. Time-related clinical outcomes were calculated from the time of peritoneal fluid collection. Clinical endpoints were evaluated in the whole study population and in the following subgroups: patients admitted to an ICU, patients hospitalized within the previous 90 days, and patients with nosocomial SBP.

Descriptive statistics were used to characterize microbiologic data in patients with culture-positive SBP. Fisher’s exact test and Mann-Whitney U test were used to compare categorical and continuous clinical outcomes, respectively. Univariate logistic regression was used to evaluate potential predictive factors for isolation of a TGC-nonsusceptible isolate, and both univariate and multivariate analyses were used to evaluate risk factors for 30- and 90-day mortality. A power analysis was conducted, and 150 patients were needed in each arm to detect a 50% reduction from a baseline mortality rate of 25%^5^ assuming an 80% power and an alpha of .05 for a two-sided test. Reported p-values represent two-sided tests. Statistical significance was considered *p* ≤ .05.

## Results

**Figure 1.**
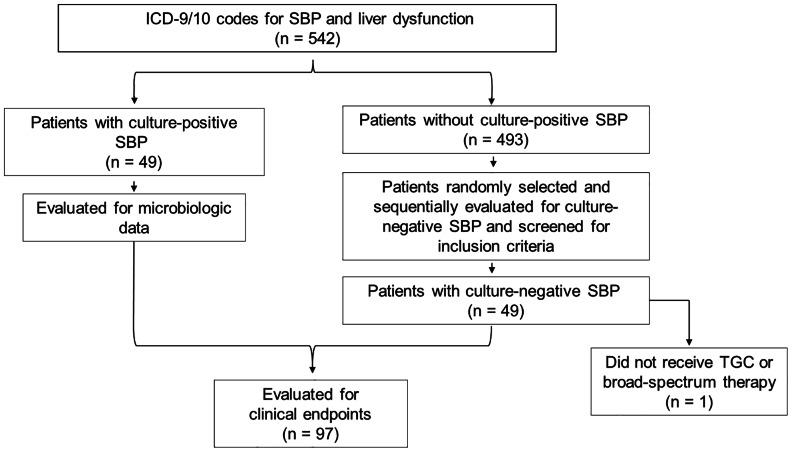
Cohort schema. SBP = spontaneous bacterial peritonitis; TGC = third-generation cephalosporin.

A total of 542 patients with an ICD-9/10 code for liver dysfunction and SBP were identified. Among these patients, 49 cases of culture-positive SBP were identified (Figure 1). Forty-nine patients with culture-negative SBP meeting inclusion criteria were initially identified. However, one patient was subsequently excluded because it was determined the patient did not receive TGC or broad-spectrum empiric antibiotic therapy. Therefore, the total study population was 97 patients (Figure 1). Baseline patient characteristics are summarized in Table 1. The median age in the entire cohort was 59 years old, and 68 (70%) patients were male. The median MELD-Na score was 25, nine (9%) patients were on SBP prophylaxis, and the most common etiologies of cirrhosis were alcoholic cirrhosis and cirrhosis of multifactorial origin. At least one proposed risk factor for MDROs was present in 80 (82%) patients, with 33% of patients admitted to the ICU, 55% hospitalized in the past 90 days, and 16% having a nosocomial infection.

**Table 1.** Baseline characteristics of whole cohort (culture-positive and culture-negative SBP). Data are expressed as *n* (%) unless specified otherwise. IQR = interquartile range; MELD = model for end-stage liver disease; SBP = spontaneous bacterial peritonitis

Baseline characteristics	All patients (N = 97)	Empiric TGC monotherapy (*n* = 58)	Empiric broad-spectrum therapy (*n* = 39)
Age—yr (median [IQR])	59 [53 – 64]	58 [52 – 63]	61 [55 – 65]
Sex, female	29 (30)	16 (27)	13 (33)
Etiology of cirrhosis			
Alcoholic cirrhosis	28 (29)	19 (33)	9 (23)
Nonalcoholic steatohepatitis	20 (20)	11 (19)	9 (23)
Hepatitis B/C	17 (17)	8 (14)	9 (23)
Other/multifactorial	32 (33)	20 (24)	12 (31)
MELD-Na score (median [IQR])	25 [21 – 30]	25 [21 – 30]	24 [22 – 30]
SBP prophylaxis at admission			
Ciprofloxacin	5 (5)	4 (7)	1 (2)
Trimethoprim/Sulfamethoxazole1	2 (2)	1 (2)	1 (2)
Norfloxacin	1 (1)	1 (2)	0 (0)
Cefdinir	1 (1)	1 (2)	0 (0)
Admitted to intensive care unit	32 (33)	14 (24)	18 (46)
Hospitalized within previous 90 days	55 (57)	30 (52)	25 (64)
Nosocomial infection	16 (16)	11 (19)	5 (13)

The most common organisms isolated in the culture-positive cohort were *Escherichia coli*, *Klebsiella pneumoniae*, and non-*pneumoniae Streptococcus* species (Table 2). Of the 49 isolates, 21 (43%) and 28 (57%) were gram-positive and gram-negative, respectively. The evaluation of MDRO prevalence in SBP at our study site demonstrated that 8 (16%) of the 49 isolates were nonsusceptible to ceftriaxone (Table 2). Among the nonsusceptible isolates, 2 organisms (one *E. coli* and one *K. pneumoniae*) were extended-spectrum beta-lactamase (ESBL) producers. None of the MDRO risk factors previously mentioned (ICU admission, recent hospitalization, and nosocomial infection) were associated with statistically significant increase in ceftriaxone-nonsusceptible isolates in a univariate analysis (Table 3).

**Table 2.** Microbiology of spontaneous bacterial peritonitis (SBP) in culture-positive SBP patients

Organism	No. of total isolates N = 49	No. of ceftriaxone-nonsusceptible isolates N = 8
*Escherichia coli*	15	1 (7%)
*Streptococcus species* (non-*pneumoniae*)	9	
*Klebsiella pneumoniae*	6	1 (17%)
*Staphylococcus aureus*		
Methicillin-susceptible	3	0 (0%)
Methicillin-resistant	1	1 (100%)
*Enterococcus faecalis*	2	2 (100%)
*Streptococcus pneumoniae*	2	
*Enterobacter cloacae*	2	1 (50%)
*Serratia marcescens*	2	
*Proteus mirabilis*	1	
*Pseudomonas aeruginosa*	1	1 (100%)
*Klebsiella oxytoca*	1	
*Listeria monocytogenes*	1	
*Microbacterium paraoxydans*	1	
*Lactobacillus* species	1	
*Enterococcus faecium*	1	1 (100%)

**Table 3.** Univariate logistic regression for the likelihood of infection with a ceftriaxone-nonsusceptible organism

Characteristic	Ceftriaxone- susceptible N = 41	Ceftriaxone- nonsusceptible N = 8	Odds ratio (OR)	95% CI	*P*-value
Admitted to ICU	15 (37%)	4 (50%)	1.73	0.36, 8.34	.5
Hospital admission in previous 90 days	22 (54%)	5 (62%)	1.44	0.31, 7.77	.6
Nosocomial infection	6 (15%)	2 (25%)	1.94	0.25, 11.1	.5

Clinical outcomes are summarized in Table 4. Fifty-eight (59%) patients were treated empirically with a TGC; 48 (83%) received ceftriaxone and 10 (17%) received cefotaxime. Thirty-five (90%) of 39 patients who received broad-spectrum empiric therapy received an anti-pseudomonal beta-lactam (20 patients received piperacillin/tazobactam, 12 patients received cefepime, 3 patients received meropenem) and 30 (76%) of 39 received therapy active against MRSA (28 patients received vancomycin, 1 patient received daptomycin, 1 patient received linezolid).

**Table 4.** Clinical outcomes of patients with SBP based on empiric therapy choice. CRO = ceftriaxone; TGC = third-generation cephalosporin; IQR = interquartile range

Outcome/characteristic	Empiric TGC monotherapy N = 58	Empiric broad-spectrum therapy N = 39	*P*-value
Culture positive—*n* (%)	32 (55)	17 (43)	.30
CRO-resistant isolate—*n* (%)	5 (9)	3 (8)	.1.00
30-day mortality—*n* (%)	21 (36)	15 (38)	1.00
90-day mortality—*n* (%)	32 (55)	22 (55)	1.00
Length of stay, days—median [IQR]	6.5 [4 – 10]	8 [5.75 – 13.25]	.25
Therapy modification—*n* (%)	21 (36)	10 (25)	.28
Due to clinical deterioration—*n* (%)	11 (19)	5 (13)	.58
Due to nonsusceptible organism identified—*n* (%)	1 (2)	2 (5)	.57
Due to other reason—*n* (%)	9 (16)	3 (8)	.35

30- and 90-day mortality were similar between patients who received empiric TGC therapy and broad-spectrum empiric therapy (Table 4). Median length of hospital stay was comparable between the TGC and broad-spectrum therapy groups (6.5 d vs 8 d; *P* = .25). There was not a statistically significant difference between the TGC and broad-spectrum therapy groups in the percentage of patients whose therapy was broadened (36% vs 25%; *P* = .28) (Table 4). No statistically significant differences in clinical outcomes were detected between patients who received empiric TGC therapy and patients who received broad-spectrum empiric therapy, including three separate subgroup analyses of patients admitted to ICUs, with recent hospital admission, and with nosocomial infections (Table 5; left, middle, and right columns, respectively).

**Table 5.** Subgroup analyses clinical outcomes based on empiric therapy choice. CRO = ceftriaxone; TGC = third-generation cephalosporin; IQR = interquartile range

Subgroup	Admission to ICU			Hospitalization in the past 90 days			Nosocomial SBP		
Outcome/characteristic	Empiric TGC monotherapy N = 14	Empiric broad-spectrum therapy N = 18	*P*-value	Empiric TGC monotherapy N = 30	Empiric broad-spectrum therapy N = 25	*P*-value	Empiric TGC monotherapy N = 11	Empiric broad-spectrum therapy N = 5	*P*-value
Culture positive—*n* (%)	10 (71)	9 (50)	.29	18 (60)	9 (36)	.11	6 (55)	2 (40)	1.00
CRO-resistant isolate—*n* (%)	2 (14)	2 (11)	1.00	3 (10)	2 (8)	1.00	1 (9)	1 (20)	.51
30-day mortality—*n* (%)	9 (64)	11 (61)	1.00	12 (40)	9 (36)	.79	3 (27)	4 (80)	.11
90-day mortality—*n* (%)	12 (86)	13 (72)	.43	19 (63)	11 (44)	.18	4 (36)	4 (80)	.28
Length of stay, days—median [IQR]	5.5 [2.25 – 9.5]	9 [7.25 – 14]	.08	8 [4 – 11]	8 [6 – 12]	.28	14 [10.5 – 19]	19 [17 – 14]	.36
Therapy modification—*n* (%)	6 (43)	5 (28)	.47	10 (33)	5 (20)	.37	5 (45)	3 (60)	1.00
Due to clinical deterioration—*n* (%)	3 (21)	2 (11)	.63	5 (17)	1 (4)	.20	3 (27)	1 (20)	1.00
Due to nonsusceptible organism identified—*n* (%)	1 (7)	1 (6)	1.00	1 (3)	2 (8)	.59	0 (0)	1 (20)	.31
Due to other reason—*n* (%)	2 (14)	2 (11)	1.00	4 (13)	2 (8)	.68	2 (18)	1 (20)	1.00

Four of the 36 patients who died by 30 days received empiric therapy to which the isolated organism was not susceptible. This included 3 patients in the TGC group whose cultures grew *E. faecalis* (2 patients) and *P. aeruginosa* and one patient in the broad-spectrum group who received empiric cefepime and vancomycin whose culture grew an ESBL-producing *E. coli*. TGC monotherapy was not associated with a higher rate of 30-day mortality in univariate or multivariate analyses; however, the presence of multiple MDRO risk factors was associated with a higher 30-day mortality rate (Table 6).

**Table 6.** Univariate and multivariate analysis of the likelihood of death within 30 days

Characteristic	Univariate			Multivariate		
	OR	95% CI	*P*-value	OR	95% CI	*P*-value
TGC monotherapy	0.91	0.39, 2.12	.8	1.18	0.48, 2.94	.7
Presence of multiple MDRO risk factors	2.46	1.33, 4.95	.007	2.52	1.34, 5.13	.006

## Discussion

Among patients hospitalized in our health system with culture-positive SBP, 84% of isolated organisms were susceptible to ceftriaxone. Susceptibility to ceftriaxone did not differ based on the presence of risk factors for MRDOs proposed by updated consensus guidelines.^4^ In this study population, 82% of patients had at least one MDRO risk factor. Providing empiric broad-spectrum therapy to all patients may be unwarranted given the low rate of ceftriaxone resistance within this health system.

Additionally, broad empiric antimicrobial selection did not appear to correlate positively or negatively with 30- or 90-day mortality. Though patients admitted to the ICU who were empirically treated with TGCs had numerically higher rates of broadening of therapy, they did not have worse clinical outcomes. While the presence of a single MDRO risk factor was not associated with higher mortality rates, patients with multiple MDRO risk factors had a statistically significant increase in 30-day mortality. Even though patients who had a ceftriaxone-nonsusceptible isolate had numerically higher mortality than patients with ceftriaxone-susceptible isolates, broad empiric therapy selection did not appear to improve outcomes in these patients. These findings suggest that inadequate activity of empiric antibiotics may not be the primary driver of mortality in SBP patients, and that, unsurprisingly, patients with multiple indicators of high-acuity illness tend to have poorer outcomes related to SBP.

This study adds to the documented phenomenon that the microbiology of SBP is highly heterogeneous with the broad range of culture-positivity rates, identified organisms, and antimicrobial resistance rates seen in different geographical locations. Studies cited in recent guidelines conducted in Italy^5^ and Korea^6^ showed ceftriaxone resistance rates of 80% and 37.5%, respectively, while a single-center study published by Sunjaya, *et al* in the US demonstrated a ceftriaxone resistance rate of only 10%.^8^ Guidelines highlight increasing rates of gram-positive organisms isolated in SBP patients, but this study and others demonstrate that most gram-positive organisms present in SBP are still adequately covered by TGCs.^8^ This degree of heterogeneity necessitates a local, institution-specific approach to microbiological monitoring, validation of proposed MDRO risk factors, and subsequent development of SBP treatment guidelines.

This retrospective evaluation has several notable limitations. Only 49 confirmed cases of culture-positive SBP were identified after microbiologic evaluation of 542 patients with an ICD-9/10 code for SBP and liver dysfunction. Most previous literature suggests culture-positivity rates for SBP of at least 30%,^6,9^ but select studies have demonstrated culture-positivity rates of <15%.^8^ The low rate of culture positivity was perplexing to the investigators. It is unlikely due to antibiotic prophylaxis, since this was present in only 9% of patients. Although unlikely, it is possible that some of the culture-negative patients who were not randomized to in-depth analysis were miscoded, which would have affected the calculation of culture-positivity rate. With this small number of culture-positive patients available, our study did not meet power to detect a relative risk reduction of 50% in the primary end point of 30-day mortality. Additionally, this study had very low rates of nosocomial SBP (17%), which is among the most frequently reported risk factors for MDRO isolation in SBP.^4^ Therefore, this study population may have lacked enough nosocomial patients to evaluate this as a MDRO risk factor. The univariate and multivariate analyses likely had insufficient quantities of patients with each individual MDRO risk factor to adequately assess each risk factor’s correlation with poor clinical outcomes. The effect of other potential mortality risk factors not included in our analyses cannot be determined.

In conclusion, this study affirms that TGCs remain a reasonable option as empiric therapy for SBP within this health system. Over 80% of organisms isolated in peritoneal fluid cultures from patients with SBP were susceptible to TGCs, and providing broader spectrum therapy to patients with at least one MDRO risk factor would necessitate giving these agents to over 80% of SBP patients. Although the outcome analysis portion of the study had limitations, including a relatively small sample size and lack of statistical power for our primary end point, a relationship between empiric broad-spectrum antimicrobial therapy and improved clinical outcomes was not found. In conjunction with previous reports of heterogeneity in SBP microbiology, this study supports the development of institution-specific protocols for empiric SBP treatment that are guided by local microbiologic characteristics.
